# A comparison between robot-assisted minimally invasive surgery and conventional craniotomy for intracerebral hemorrhage: efficiency, complications and outcomes

**DOI:** 10.3389/fneur.2025.1688309

**Published:** 2026-01-09

**Authors:** Mingxiao Li, Yufei Gao, Ce Wang, Aimin Li, Ziyi Liu, Yulian Zhang, Lijun Yang, Yanbing Yu, Xueke Zhen

**Affiliations:** 1 Department of Neurosurgery, China-Japan Friendship Hospital, Beijing, China; 2 Department of Neurosurgery, The Second Hospital of Hebei Medical University, Shijiazhuang, China

**Keywords:** intracerebral hemorrhage, robot-assisted minimally invasive surgery, craniotomy, hematoma evacuation, prognosis

## Abstract

**Background:**

Intracerebral hemorrhage (ICH) is a severe neurological condition with high morbidity and mortality rates. Robot-Assisted Minimally Invasive Surgery (RA-MIS) has emerged as a novel technique that may offer advantages over traditional craniotomy. This study aims to evaluate the clinical efficacy of RA-MIS compared to conventional craniotomy in patients with ICH.

**Methods:**

A retrospective cohort study was conducted involving 44 patients with ICH admitted to two medical centers between December 1, 2022, and October 31, 2024. Patients were divided into two groups: 24 underwent RA-MIS, and 20 underwent traditional craniotomy. Baseline characteristics, functional outcomes [modified Rankin Scale (mRS)], neurological deficits [National Institutes of Health Stroke Scale (NIHSS)], postoperative complications, hospitalization costs, duration of respiratory support, and mortality rates were analyzed.

**Results:**

The RA-MIS group demonstrated significantly better functional outcomes at 90 days postoperative, with a mean mRS score of 2.58 ± 1.72 compared to 3.85 ± 1.63 in the craniotomy group (*P* = 0.017). NIHSS scores at 90 days were also significantly lower in the RA-MIS group (3.64 ± 3.32 vs. 7.71 ± 5.35; *P* = 0.006), indicating improved neurological recovery. RA-MIS patients experienced fewer postoperative complications, including lower incidences of pneumonia (16.7% vs. 70.0%; *P* < 0.001) and intracranial infections (0.0% vs. 20.0%; *P* = 0.036). The total hospitalization costs were significantly lower for the RA-MIS group (¥78,677 ± 38,904 vs. ¥136,399 ± 85,916; *P* = 0.006), and the duration of respiratory support was shorter (64.00 ± 161.79 h vs. 238.25 ± 197.04 h; *P* = 0.002). The mortality rate was significantly lower in the RA-MIS group (8.3% vs. 30.0%; *P* = 0.020).

**Conclusions:**

RA-MIS is associated with improved functional and neurological outcomes, fewer postoperative complications, reduced hospitalization costs, and lower mortality rates compared to traditional craniotomy in patients with ICH. These findings suggest that RA-MIS may be a more effective and economical surgical option for hematoma evacuation in ICH patients.

## 1 Introduction

Intracerebral hemorrhage (ICH), defined as the acute extravasation of blood into the brain parenchyma from a ruptured vessel, can lead to irreversible brain injury and poor outcomes. The global incidence of ICH is estimated to be ~24.6 cases per 100,000 person-years, underscoring its significant worldwide health burden. ICH accounts for ~10%−15% of all strokes, with an estimated mortality rate of 30%−40% (1). Major causes of ICH in adults include hypertension, cerebral amyloid angiopathy, and anticoagulation therapy. The condition can progress rapidly, often resulting in severe neurological deficits or death (2). Survivors frequently suffer from disabilities and face a high risk of recurrent stroke, cognitive decline, and systemic complications (3). Therefore, effective treatment protocols and comprehensive critical care are essential to improve prognosis.

Over the past decades, understanding of ICH has advanced, and numerous treatment strategies have been explored to enhance patient outcomes (4). These include conservative approaches such as aggressive blood pressure reduction and administration of hemostatic agents like tranexamic acid or recombinant activated factor VII. Surgical interventions, including conventional craniotomy with or without hematoma drainage (5), image-guided stereotactic endoscopic aspiration (6–8), and minimally invasive catheter evacuation followed by thrombolysis, have also been employed (9). Decompressive craniectomy, a conventional open procedure, involves the removal of a large bone flap to relieve intracranial pressure and facilitate hematoma evacuation under direct visualization.

The development of minimally invasive surgery (MIS) has introduced novel intervention options that accelerate hematoma clearance and substantially improve prognoses (10, 11). Robot-assisted MIS (RA-MIS) leverages imaging data to provide accurate, individualized, and optimal surgical pathways for hematoma evacuation with minimal damage to surrounding tissue (12). This approach is particularly beneficial for hematomas located in critical brain structures such as the basal ganglia, thalamus, cerebellum, and brainstem, as RA-MIS allows for precise surgical planning to avoid injury to vital vessels, nerve tracts, and nuclei (13). This study aims to investigate the clinical efficacy of RA-MIS for intracranial hematoma and to perform a systematic comparison between RA-MIS and open craniotomy in a real world.

## 2 Methods

### 2.1 Study population

A consecutive cohort of patients with intracerebral hemorrhage admitted to the Departments of Neurosurgery at China-Japan Friendship Hospital and The Second Hospital of Hebei Medical University between December 1, 2022, and October 31, 2024, were included in this study. All patients were diagnosed with intracranial hematoma according to the Guidelines for Cerebral Hemorrhage in China (2019) and received surgical intervention. Patients were included if they had a confirmed diagnosis of ICH and underwent either RA-MIS or traditional craniotomy. Exclusion criteria were: (1) subarachnoid or intraventricular hemorrhage; (2) ICH due to moyamoya disease, cerebral amyloid angiopathy, aneurysm, or arteriovenous malformation; (3) recent history of trauma; (4) presence of malignancies or other life-threatening diseases; and (5) unstable vital signs at admission. Demographic, clinical, radiological, and prognostic data were collected from medical records for analysis. This study was approved by the institutional review board of China-Japan Friendship Hospital and The Second Hospital of Hebei Medical University (Approved ID: 2024-R147). All methods were in accordance with the principles of the Declaration of Helsinki. The signed consent forms, including for publication of identifying information/images in an online open-access publication, were received from all subjects and/or their legal guardian(s). As this was a retrospective cohort study, the sample size was determined by the consecutive enrollment of all eligible patients during the study period, and no pre-study sample size calculation was performed.

### 2.2 Radiological assessment

All patients underwent emergency non-contrast head computed tomography (CT) scans (GE Discovery CT750HD, USA; 120 kV, 400 mA, slice thickness 1 mm, gap 0.1 mm) to determine the location and volume of the hematomas. Hematoma locations were classified as cerebral hemisphere, basal ganglia/thalamus, or infratentorial structures (brainstem or cerebellum) based on the geographic epicenter. Hematoma volumes were calculated using the ABC/2 method, where A is the largest diameter of the hemorrhage, B is the diameter perpendicular to A on the same slice, and C is the number of 1-mm slices containing the hemorrhage (14).

### 2.3 Surgical procedures

#### 2.3.1 Robot-assisted minimally invasive surgery (RA-MIS)

**Figure 1:**
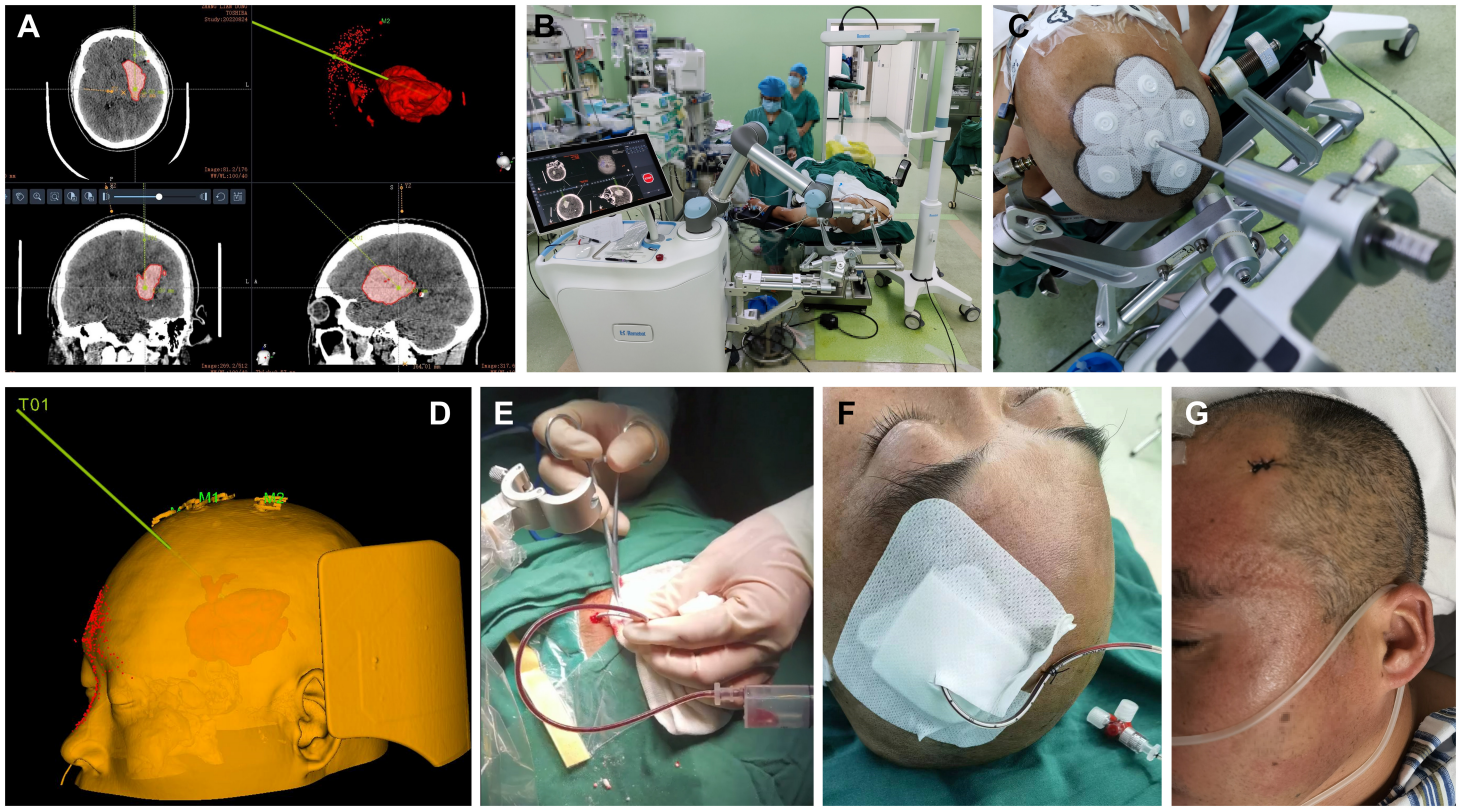
Comparison of outcomes between RA-MIS and Craniotomy Groups. **(A)** Comparison of residual hematoma volumes at discharge between RA-MIS (4.79 ± 6.62 mL) and craniotomy (4.20 ± 5.45 mL) groups, indicating effective hematoma evacuation by both surgical methods (*P* = 0.754). **(B)** Comparison of hospital stay duration, with significantly shorter stays observed in the RA-MIS group (16.92 ± 12.51 days) compared to the craniotomy group (27.06 ± 20.13 days; *P* = 0.046). **(C)** Total hospitalization costs were significantly lower in the RA-MIS group (¥78,677 ± 38,904) than in the craniotomy group (¥136,399 ± 85,916; *P* = 0.006). **(D)** Incidence of postoperative complications, with pneumonia occurring less frequently in the RA-MIS group (16.7%) compared to the craniotomy group (70.0%; *P* < 0.001). There were also differences in tracheotomy rates (12.5% in RA-MIS vs. 55.0% in craniotomy; *P* = 0.004). No significant differences were noted between groups for deep venous thrombosis, acute renal failure, stress ulcers, or hydrocephalus. **(E)** Kaplan–Meier survival curve showing cumulative mortality rates, with significantly lower mortality in the RA-MIS group (8.3%) compared to the craniotomy group (30.0%; *P* = 0.020). **(F)** Comparison of mean mRS scores at discharge, showing a trend toward better functional outcomes in the RA-MIS group (3.29 ± 1.628) compared to the craniotomy group (3.90 ± 1.210; *P* = 0.174). **(G)** Sankey diagram showing changes in mRS scores for the craniotomy group at baseline, discharge, and 90 days postoperative. **(H)** Changes in mRS scores for the RA-MIS group at baseline, discharge, and 90 days postoperative.

Patients and their families were provided with detailed explanations of both RA-MIS and traditional craniotomy and chose their preferred treatment. For those opting for RA-MIS, individualized surgical paths were designed by two senior neurosurgeons (CW and XZ) using the latest imaging data (example in Figure 1A). Under general anesthesia, patients were placed in the supine position, and standard sterile preparation was performed (Figure 1B). During surgery, patients were co-registered with the robotic system to map the planned trajectory from image space to real space (Figure 1C). Guided by the surgical robot, the entry point was determined (Figure 1D), and a small incision (~2 cm) was made. A burr hole (5 mm) was drilled along the robot-determined trajectory, carefully avoiding critical vessels and critical brain structures. A ventricular catheter was then advanced to the target depth within the hematoma under robotic guidance. Aspiration was performed slowly, considering intracranial pressure and hematoma characteristics (Figure 1E). After catheter placement, the incision was closed in layers (Figure 1F).

Postoperative CT scans were performed 4–6 h later to confirm catheter positioning and rule out new bleeding. Urokinase (50,000 IU; Livzon, Zhuhai, Guangdong, China) was administered into the hematoma cavity every 12 h for up to nine doses or until the residual hematoma volume was ≤ 10 cm3. After each dose, the catheter was clamped for 2 h and then reopened for gravity drainage for 10 h. The catheter and intracranial pressure monitor were typically removed within 6 days.

#### 2.3.2 Traditional craniotomy

For patients undergoing traditional craniotomy, conventional craniotomy was performed via a fronto-temporo-parietal approach. A large bone flap measuring at least 10 × 10 cm^2^ was removed to allow adequate hematoma evacuation and cerebral decompression. The dura mater was carefully opened to expose the hematoma cavity. Hematoma evacuation was performed under direct visualization using microsurgical techniques to minimize damage to surrounding brain tissue. After complete evacuation of the hematoma, a drainage catheter was placed in the cavity to allow for postoperative drainage of residual blood and cerebrospinal fluid. Hemostasis was meticulously achieved to prevent postoperative re-bleeding. The dura mater was left open or loosely approximated to accommodate brain swelling. The surgical site was closed in layers: the galea aponeurotica and subcutaneous tissues were sutured using absorbable sutures, and the skin was closed with non-absorbable sutures or staples. The bone flap was not immediately replaced to allow for decompression; it was preserved for potential future cranioplasty.

### 2.4 Postoperative management and follow-up

All patients received standard medical management according to the Chinese Guidelines for the Diagnosis and Treatment of Cerebral Hemorrhage (2022) (15). Dehydrating agents such as mannitol, furosemide, and glycerin fructose were administered to alleviate cerebral edema. Common postoperative complications, including pneumonia, intracranial infection, and the need for tracheotomy (see Results section 3.3 for frequencies), were managed proactively under the guidance of senior physicians according to established clinical guidelines.

Patients were followed up every 3–4 months via telephone or as clinically indicated. Functional outcomes were assessed using the modified Rankin Scale (mRS) and the National Institutes of Health Stroke Scale (NIHSS) (16). Overall survival (OS) was defined as the time from admission to death or the last follow-up date.

### 2.5 Statistical analysis

Continuous variables were analyzed using the Student's *t*-test or the Mann–Whitney *U*-test, depending on data distribution. Categorical variables were compared using the Chi-square test or Fisher's exact test. Survival analysis was performed using Kaplan-Meier curves and the log-rank test. Statistical analyses were conducted using GraphPad Prism 9.0.1 (GraphPad Software, USA) and SPSS version 25.0 (IBM Corporation, Armonk, NY, USA). A two-sided *P*-value of <0.05 was considered statistically significant.

## 3 Results

### 3.1 Demographic, clinical, and radiological characteristics

A total of 44 patients were included in the study, with 24 patients undergoing Robot-Assisted Minimally Invasive Surgery (RA-MIS) and 20 patients receiving craniotomy. The mean age was similar between the RA-MIS group (58.50 ± 13.47 years) and the craniotomy group (59.55 ± 11.15 years; *P* = 0.782). Gender distribution did not differ significantly, with females comprising 45.8% of the RA-MIS group and 35.0% of the craniotomy group (*P* = 0.547, Table 1).

**Table 1:** Baseline characteristics of RA-MIS and craniotomy treatment patients.

**Characteristics**	**Total (*N* = 44)**	**RA-MIS (*N* = 24)**	**Craniotomy (*N* = 20)**	***P*-value**
Age (yrs, mean ± SD)	58.98 ± 12.34	58.50 ± 13.47	59.55 ± 11.15	0.782
**Gender**				
Female (*n*, %)	18 (40.9)	11 (45.8)	7 (35.0)	0.547
Male (*n*, %)	26 (59.1)	13 (54.2)	13 (65.0)	
**Location**				
Cerebral hemisphere	7 (15.9)	2 (8.3)	5 (25.0)	0.306
Basal ganglia/Thalamus	34 (77.3)	20 (83.3)	14 (70.0)	
Infratentorial structure	3 (6.8)	2 (8.3)	1 (5)	
**Hypertension (** ***n*** **, %)**				
Yes	30 (68.2)	19 (79.2)	11 (55.0)	0.112
No	14 (31.8)	5 (20.8)	9 (45.0)	
**Diabetes (** ***n*** **, %)**				
Yes	13 (29.5)	7 (29.2)	6 (30.0)	1.000
No	31 (70.5)	17(70.8)	14 (70.0)	
GCS at admission (mean ± SD)	8.91 ± 3.49	9.61 ± 0.67	8.20 ± 0.84	0.199
HV at admission (mean ± SD)	49.23 ± 28.13	41.72 ± 24.43	58.23 ± 30.19	0.057
mRS at admission (mean ± SD)	4.20 ± 0.98	4.00 ± 0.95	4.45 ± 1.00	0.140
NHISS at admission (mean ± SD)	10.70 ± 4.24	11.22 ± 1.27	10.95 ± 0.99	0.869

GCS, Glasgow Coma Scale; HV, hematoma volumes; mRS, modified rankin scale; NIHSS, National Institutes of Health Stroke Scale.

Hematoma locations were predominantly in the basal ganglia or thalamus for both groups (RA-MIS: 83.3%; craniotomy: 70.0%; *P* = 0.306). Hypertension was more prevalent in the RA-MIS group (79.2%) compared to the craniotomy group (55.0%), although this difference was not statistically significant (*P* = 0.112). The incidence of diabetes mellitus was nearly identical between the RA-MIS group (29.2%) and the craniotomy group (30.0%; *P* = 1.000, Table 1).

At admission, the mean Glasgow Coma Scale (GCS) score was slightly higher in the RA-MIS group (9.61 ± 0.67) than in the craniotomy group (8.20 ± 0.84), but this difference did not reach statistical significance (*P* = 0.199). The mean hematoma volume at admission tended to be lower in the RA-MIS group (41.72 ± 24.43 mL) compared to the craniotomy group (58.23 ± 30.19 mL), approaching statistical significance (*P* = 0.057). The mean modified Rankin Scale (mRS) scores at admission were 4.00 ± 0.95 for RA-MIS patients and 4.45 ± 1.00 for craniotomy patients (*P* = 0.140). National Institutes of Health Stroke Scale (NIHSS) scores were comparable between the groups (RA-MIS: 11.22 ± 1.27 *vs*. craniotomy: 10.95 ± 0.99; *P* = 0.869, Table 1).

### 3.2 Comparison of hospitalization costs, respiratory support duration, and hospital stay

**Figure 2:**
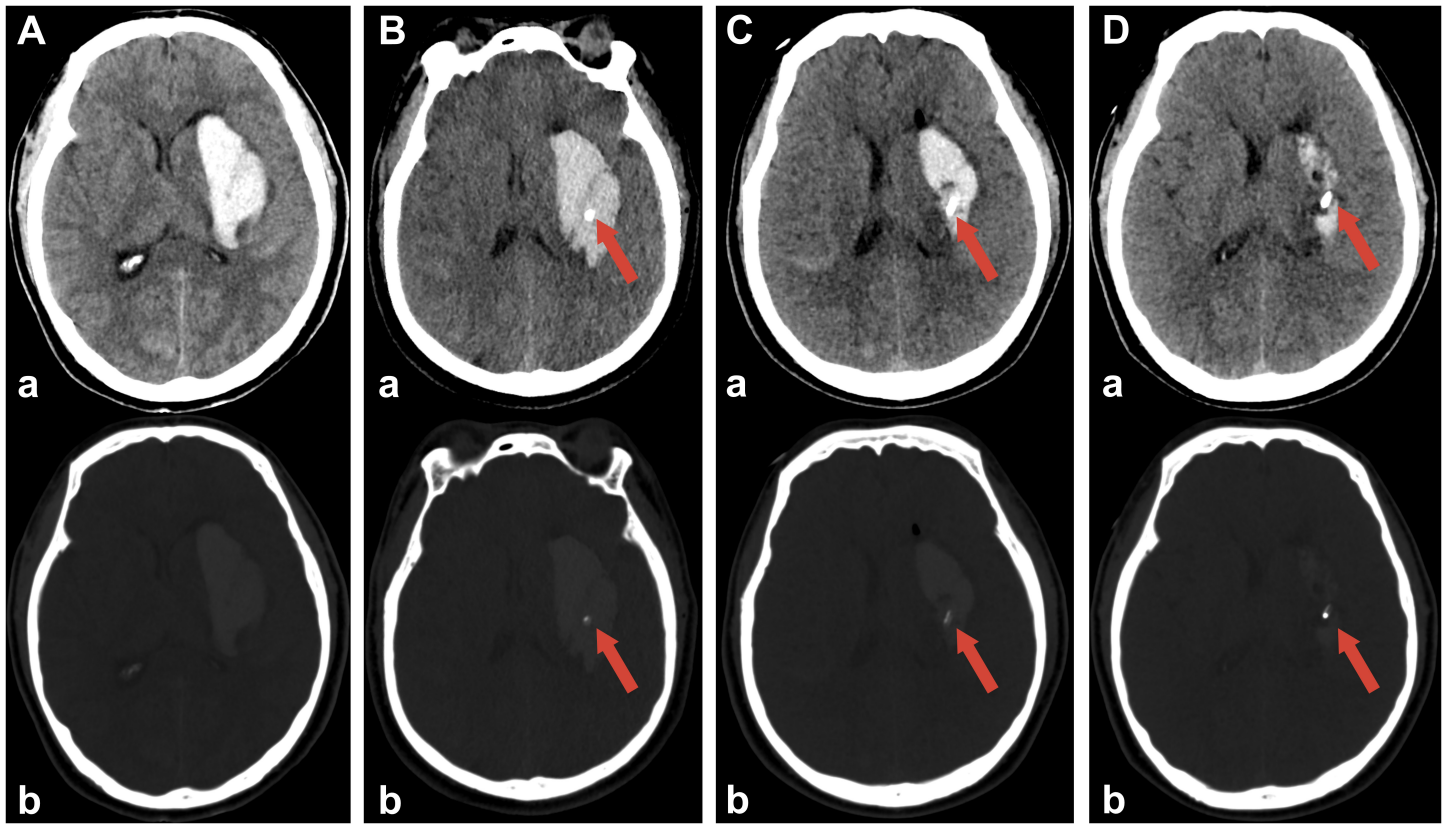
Representative case of disease dynamic change in the craniotomy group. **(A)** Preoperative CT scan showing the intracerebral hematoma in right basal ganglia. **(B)** Postoperative CT scan showing near-complete hematoma evacuation. **(C)** CT scan 3 days post-operation showing continued resolution of the hematoma. **(D)** CT scan 14 days post-surgery demonstrating further recovery and stabilization, mild brain bulging is still present. Red arrows indicate the placement of the drainage catheter in each scan, with ‘a' for non-contrast images and ‘b' for bone window.

**Figure 3:**
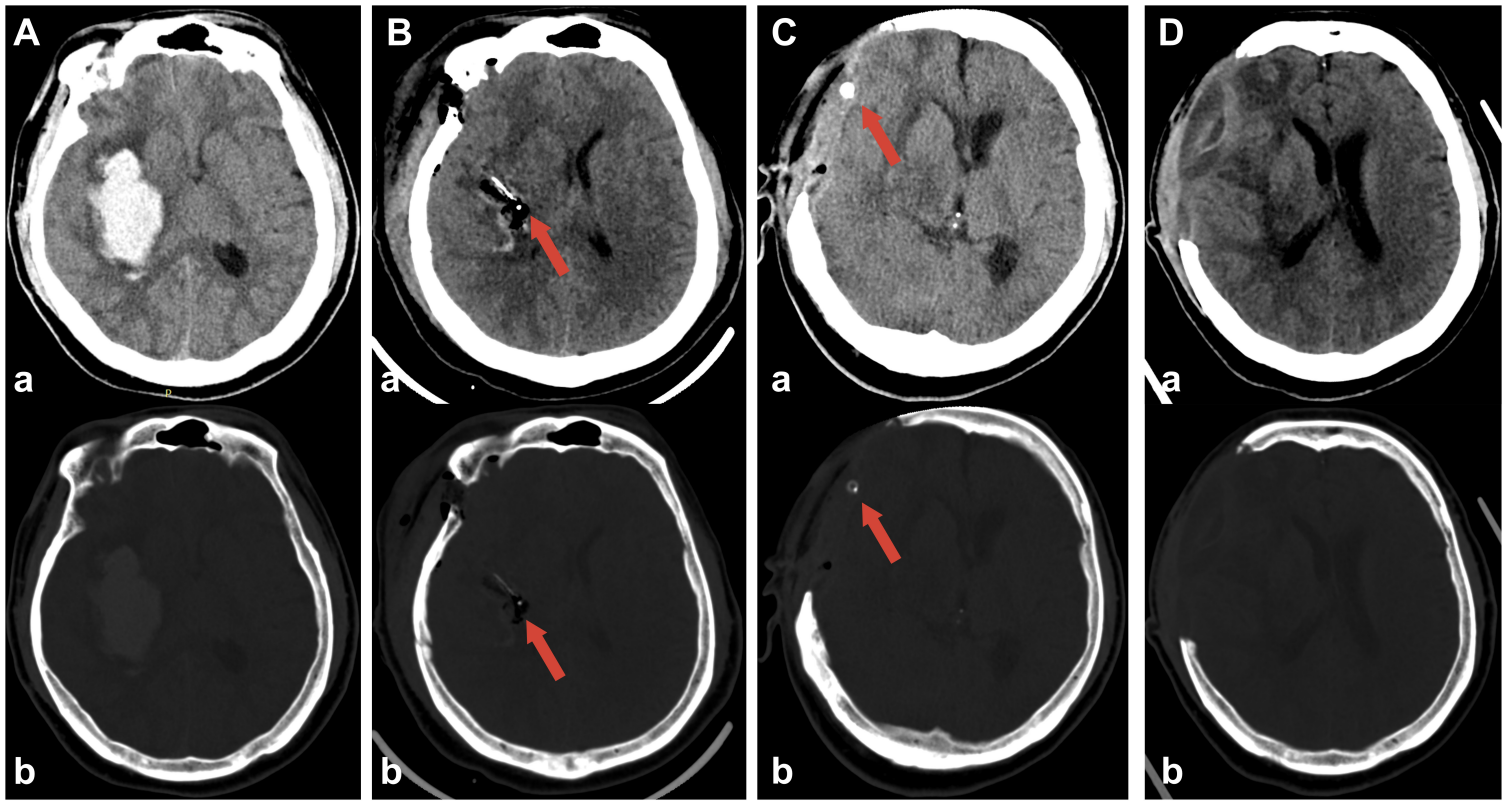
Representative case demonstrating dynamic changes in a patient treated with RA-MIS. **(A)** Preoperative CT scan showing intracerebral hemorrhage in the left basal ganglia. **(B)** Four hours after robot-assisted minimally invasive surgery, the CT scan confirms successful placement of the drainage catheter into the hematoma cavity. **(C)** Following urokinase injection into the hematoma cavity, accumulated blood was successfully evacuated; the CT scan at 24 h post-operation shows a significant reduction in the hematoma size. **(D)** CT scan at 3 days post-operation demonstrating near-complete resolution of the hematoma. Red arrows indicate the placement of the drainage catheter in each scan, with ‘a' for non-contrast images and ‘b' for bone window.

**Figure 4:**
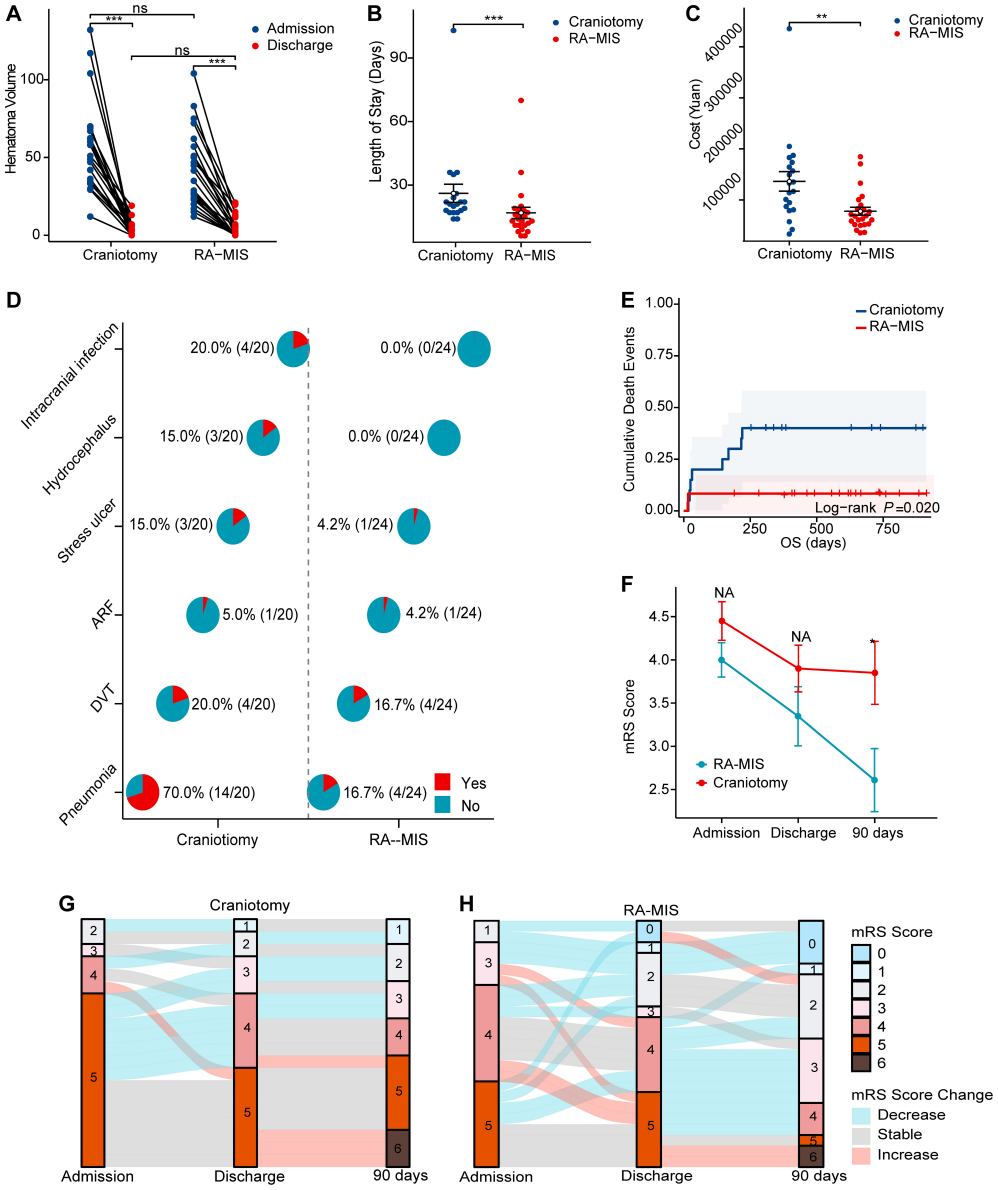
A series of graphs comparing craniotomy and RA-MIS. **(A)** Line graph showing hematoma volume changes from admission to discharge. **(B)** Scatter plot depicting length of stay with RA-MIS having shorter durations. **(C)** Scatter plot of costs showing RA-MIS as less expensive. **(D)** Pie charts representing complications like infection, highlighting fewer in RA-MIS. **(E)** Kaplan-Meier curves showing cumulative death events, indicating better survival for RA-MIS. **(F)** Line chart of modified Rankin Scale (mRS) scores, RA-MIS shows improvement over time. **(G, H)** Sankey diagrams for mRS score transitions from admission to 90 days, RA-MIS shows more improvements.

At discharge, the mean residual hematoma volumes were similar between the two groups (RA-MIS: 4.79 ± 6.62 mL *vs*. craniotomy: 4.20 ± 5.45 mL; *P* = 0.754), indicating effective hematoma evacuation by both surgical methods (illustrative examples are shown in Figure 2 for craniotomy and Figure 3 for RA-MIS; results comparison in Figure 4A). Importantly, the average length of hospital stay was significantly reduced in the RA-MIS group. The overall mean hospital stay for all patients was 21.07 ± 16.63 days. Patients in the RA-MIS group had a shorter hospital stay of 16.92 ± 12.51 days compared to 27.06 ± 20.13 days in the craniotomy group (*P* = 0.046, Figure 4B). This reduction in hospitalization duration suggests that RA-MIS may facilitate faster postoperative recovery and discharge. The total hospitalization costs were also significantly lower for patients in the RA-MIS group (¥78,677 ± 38,904) compared to those in the craniotomy group (¥136,399 ± 85,916; *P* = 0.006, Figure 4C). Additionally, the length of respiratory support required was significantly shorter for the RA-MIS group (64.00 ± 161.79 h) than for the craniotomy group (238.25 ± 197.04 h; *P* = 0.002, Table 2).

**Table 2:** Outcomes of patients who received RA-MIS and craniotomy.

**Outcomes**	**Total (*N* = 44)**	**RA-MIS (*N* = 24)**	**Craniotomy (*N* = 20)**	***P*-value**
**mRS**				
mRS at admission (mean ± SD)	4.20 ± 0.98	4.00 ± 0.95	4.45 ± 1.00	0.140
mRS at discharge	3.57 ± 1.469	3.29 ± 1.628	3.90 ± 1.210	0.174
mRS at 90 days postoperative	3.16 ± 1.778	2.58 ± 1.717	3.85 ± 1.631	0.017
NHISS at admission (mean ± SD)	10.70 ± 4.24	11.22 ± 1.27	10.95 ± 0.99	0.869
NHISS at discharge	8.30 ± 5.587	6.78 ± 5.33	10.05 ± 5.48	0.055
NHISS at 90 days postoperative	5.41 ± 4.72	3.64 ± 3.32	7.71 ± 5.35	**0.006** ^*^
Length of stay (days, mean ± SD)	21.07 ± 16.63	16.92 ± 12.51	27.06 ± 20.13	**0.046** ^*^
Total costs (Yuan, mean ± SD)	105524 ± 70574	78677 ± 38904	136399 ± 85916	**0.006** ^*^
Length of respiratory support	143.20 ± 197.13	64.00 ± 161.79	238.25 ± 197.04	**0.002** ^*^
HV at admission (mean ± SD)	49.23 ± 28.13	41.72 ± 24.43	58.23 ± 30.19	0.057
HV at discharge (mean ± SD)	4.51 ± 6.03	4.79 ± 6.62	4.20 ± 5.45	0.754
**Complications (%**, ***n*****/*****N*****)**				
Pneumonia	40.9 (18/44)	16.7 (4/24)	70.0% (14/20)	<0.001^*^
Deep venous thrombosis	18.2 (8/44)	16.7 (4/24)	20.0% (4/20)	1.00
Acute renal failure	4.5 (2/44)	4.2 (1/24)	5.0(1/20)	1.00
Stress ulcer	9.1 (4/44)	4.2 (1/24)	15.0 (3/20)	0.316
Hydrocephalus	6.8 (3/44)	0.0 (0/24)	15.0 (3/20)	0.086
Intracranial infection	9.1 (4/44)	0.0 (0/24)	20.0 (4/20)	**0.036** ^*^
Tracheotomy	31.8 (14/44)	12.5 (3/24)	55.0 (11/20)	**0.004** ^*^

GCS, Glasgow Coma Scale; HV, hematoma volumes; mRS, Modified Rankin Scale; NIHSS, National Institutes of Health Stroke Scale. The asterisk (^*^) indicates a statistically significant difference.

### 3.3 Postoperative complications

The incidence of postoperative complications differed notably between the groups. Pneumonia occurred significantly less frequently in the RA-MIS group (16.7%) compared to the craniotomy group (70.0%; *P* < 0.001, Figure 4D). Intracranial infections were absent in the RA-MIS group but were observed in 20.0% of patients in the craniotomy group (*P* = 0.036). The need for tracheotomy was significantly lower in the RA-MIS group (12.5%) compared to the craniotomy group (55.0%; *P* = 0.004). There were no significant differences between the groups regarding the incidence of deep venous thrombosis (RA-MIS: 16.7% vs. craniotomy: 20.0%; *P* = 1.000), acute renal failure (RA-MIS: 4.2% vs. craniotomy: 5.0%; *P* = 1.000), stress ulcers (RA-MIS: 4.2% vs. craniotomy: 15.0%; *P* = 0.316), or hydrocephalus (RA-MIS: 0.0% vs. craniotomy: 15.0%; *P* = 0.086, Figure 4D).

### 3.4 Functional and neurological outcomes at discharge and 90-day follow-up

Survival analysis revealed that the cumulative mortality rate was significantly lower in the RA-MIS group. By the last follow-up, a total of eight patients had died: 2 out of 24 patients in the RA-MIS group (8.3%) and 6 out of 20 patients in the craniotomy group (30.0%), indicating a more favorable prognosis for RA-MIS patients (*P* = 0.020, Figure 4E).

Upon discharge, the RA-MIS group showed a trend toward better functional outcomes, with a mean mRS score of 3.29 ± 1.628 compared to 3.90 ± 1.210 in the craniotomy group (*P* = 0.174, Figure 4F). At 90 days postoperative, the RA-MIS group had a significantly lower mean mRS score (2.58 ± 1.717) than the craniotomy group (3.85 ± 1.631; *P* = 0.017, Figures 4F–H), indicating improved long-term functional recovery. In terms of neurological deficits, the RA-MIS group exhibited lower NIHSS scores at discharge (6.78 ± 5.33) compared to the craniotomy group (10.05 ± 5.48), approaching statistical significance (*P* = 0.055). This improvement became significant at 90 days postoperative, with the RA-MIS group achieving a mean NIHSS score of 3.64 ± 3.32 vs. 7.71 ± 5.35 in the craniotomy group (Figures 4G, H; *P* = 0.006, Table 2).

## 4 Discussion

In this study, we evaluated the clinical efficacy of RA-MIS compared to traditional craniotomy in patients with ICH. Our findings indicate that RA-MIS is associated with better functional outcomes, reduced neurological deficits, lower hospitalization stay and costs, shorter durations of respiratory support, and fewer postoperative complications. These results align with and extend upon recent literature, suggesting that RA-MIS may offer significant advantages over conventional surgical methods for hematoma evacuation in ICH patients (17–20).

Recent advancements in minimally invasive surgical techniques have shown promising results in the management of ICH. The Minimally Invasive Surgery plus Recombinant Tissue-type Plasminogen Activator for Intracerebral Hemorrhage Evacuation (MISTIE) III trial demonstrated that minimally invasive catheter evacuation followed by thrombolysis significantly reduced hematoma volume and modestly improved functional outcomes compared to standard medical care (21). Our study builds upon these findings by incorporating robotic assistance, which enhances precision and may further improve patient outcomes.

Moreover, a recent meta-analysis concluded that minimally invasive surgery (MIS) for ICH is associated with lower mortality and better functional outcomes compared to conventional craniotomy (22). Our results corroborate these conclusions, showing that RA-MIS patients had significantly lower mortality rates and better functional recovery as measured by mRS and NIHSS scores.

Robotic assistance in neurosurgery has been gaining attention due to its potential to improve surgical accuracy and patient safety. A study by Wu et al. (23) reported that robot-assisted endoscopic surgery for ICH evacuation resulted in higher hematoma clearance rates and better neurological outcomes compared to traditional methods. Similarly, our study found that RA-MIS not only effectively evacuated hematomas but also led to superior functional and neurological outcomes.

Our RA-MIS group exhibited significantly better functional outcomes at 90 days postoperative, with a significant lower mRS score compared to the craniotomy group. This finding is consistent with the results of Hanley et al. (21), who reported that patients undergoing MIS had better functional status at 365 days. The enhanced recovery in our RA-MIS group may be attributed to reduced surgical trauma and preservation of neural structures, as suggested by other studies emphasizing the benefits of minimally invasive approaches (24, 25).

In terms of neurological deficits, the RA-MIS group showed significantly lower NIHSS scores at 90 days postoperative, indicating better neurological recovery. This aligns with findings from Hannah et al. (26), who observed improved neurological function in patients treated with MIS compared to those receiving conservative treatment. The precision of robotic assistance likely contributes to minimizing additional neurological damage during surgery.

Postoperative complications can significantly impact patient recovery and healthcare costs. Our study demonstrated that RA-MIS patients experienced fewer complications, particularly pneumonia and intracranial infections. These results are in line with a study by Tang et al. (27), which reported lower complication rates in patients undergoing MIS for ICH. The reduced invasiveness of RA-MIS likely contributes to decreased infection risk and faster mobilization, thereby lowering pneumonia incidence.

The economic implications of surgical interventions are critical, especially in resource-limited settings. Our findings indicate that RA-MIS is associated with significantly lower hospitalization costs (¥78,677 ± 38,904, ~10,900 ± 5,400 USD) compared to craniotomy (¥136,399 + 85,916, ~18,900 ± 11,900 USD). This cost reduction is supported by Zhong et al. (12), who found that MIS led to decreased hospital stays and lower overall expenses. The shorter duration of respiratory support and reduced need for ICU care in our RA-MIS group further contribute to cost savings.

RA-MIS offers several technical advantages over traditional surgical methods. The use of robotic systems allows for precise planning and execution of surgical trajectories, reducing the risk of damaging critical brain structures. This is particularly important for hematomas located in deep or eloquent brain areas (28). Tan et al. (9) emphasized that robotic assistance enhances the accuracy of hematoma evacuation, leading to better patient outcomes (19). Our study supports this notion, demonstrating that RA-MIS patients had better functional and neurological recovery.

Despite the encouraging results, our study has limitations that warrant consideration. The relatively small sample size may limit the generalizability of our findings. However, the significant differences observed between groups suggest a meaningful impact of RA-MIS on patient outcomes. Future studies with larger cohorts are necessary to confirm these results. Additionally, the non-randomized design introduces potential selection bias. Patients chose their treatment modality after being informed of the options, which may influence outcomes. Randomized controlled trials are needed to eliminate such biases and provide higher-level evidence.

Our study adds to the growing body of evidence supporting the use of MIS, particularly RA-MIS, in the management of ICH. The significant improvements in functional outcomes, neurological recovery, and reduced complications suggest that RA-MIS should be considered a viable first-line surgical option for eligible patients. As robotic technology becomes more accessible, its integration into neurosurgical practice could standardize care and improve outcomes on a broader scale.

To further validate the benefits of RA-MIS, multicenter randomized controlled trials with larger sample sizes are necessary. Long-term follow-up studies assessing quality of life and functional independence are also important to understand the sustained impact of RA-MIS. Additionally, cost-effectiveness analyses incorporating direct and indirect costs will provide valuable insights for healthcare policymakers.

Advancements in robotic technology, such as integration with real-time imaging and artificial intelligence, may enhance surgical precision and outcomes (29–34). Research exploring these innovations could further solidify the role of RA-MIS in neurosurgery.

## 5 Conclusion

In conclusion, our study demonstrates that RA-MIS is a safe and effective surgical option for patients with intracerebral hemorrhage. Compared to traditional craniotomy, RA-MIS offers superior functional and neurological outcomes, fewer postoperative complications, and economic benefits. These findings, in line with recent literature, support the adoption of RA-MIS as a preferred surgical approach in the appropriate clinical context. Further research is needed to confirm these results and facilitate the widespread implementation of robotic-assisted neurosurgical techniques.

## Data Availability

The raw data supporting the conclusions of this article will be made available by the authors, without undue reservation.
